# Mental Rest

**Published:** 1880-07

**Authors:** 


### MENTAL REST.

When a locomotive is under full headway it can not be safely stopped in a moment; the stream of steam must be gradually turned in another direction, and made to play on thin air, or on the fly-wheel, as well as to have its supply cut off. So when the nervous energy of the human system has been acting on the brain under a "full head" for an hour or more, as in the performance of the most harrowing tragedy, or in the delivery of an impassioned address, or in the execution of some momentous surgical operation, it is not safe to arrest instantly the outgoing of that power through the brain; the fact is, it is not possible if the performers just named were carried direct from the theater of their operations to a prison or vacant room, and were so bound that bodily motion was impossible, the mind would run in ceaseless circles over the performances, would be vainly striking against the air, and sleep would be impossible, except as a result of sheer exhaustion; even then it would not bring its natural renovation; the tragedian, in spite of himself, would go over his part; the orator would rehearse his sentences; the advocate would joint together again his points and proofs; the minister repeat his weighty appeals; and the surgeon perform again his terrible operations, all in the mind, vainly, and with the almost invariable accompaniment, disagreeable and wearing—to wit, measuring the effects which might have resulted from certain variations in their respective performances, the surgeon would think that his operation might have been sooner performed, or would have had a more favorable recovery if he had done this, that, or the other thing which he had not done; the clergyman will have his conscience touched by the reflection that if he had applied another text of Scripture, or presented another line of argument, or had summoned a deeper feeling of the heart, his discourse would have made a more lasting impression, and might have eventuated in more ineffaceable convictions. In one sense, these are vain thoughts; they increase the exhaustion attendant on the previous actual labors, and are altogether unprofitable. The greatest lady tragedienne of modern times, Rachel, after an exciting performance, would go home, and although past midnight, would sometimes spend an hour or more in the physical effort of moving the furniture of one room into another, and in arranging it, as if it were to remain so for months, as a means of calming the mental excitement, so that she could go to sleep; the philosophy of the matter was that the nervous energy was diverted from the brain, and compelled, in a measure, to pass out of the system through muscular action, while the mental exercise necessary was such as to engage a different portion of the brain altogether, allowing those organs opportunity of quiescence, which had been so lately exercised to an unwonted degree. Our clerical readers know it often happens that Sunday night is the worst night for sleep in the week, especially for those lazy and improvident and unsystematic unfortunates who put off their preparation for the Sabbath until the very last moment, as it were, and hence have to sit up late on Saturday night, and even encroach on the sacred hours of the Sabbath, thus profaning holy time, in the feeling that the end sanctifies the means, or that it is a perfectly legitimate labor, forgetting that it is an unnecessary labor, as it might and ought to have been done in proper work-days. As we were saying, clergymen sometimes can not get to sleep for hours after preaching at night; let such take a lesson from the above recital, and instead of going to bed as soon as they get home, let them perform some muscular movements, with the end above named in view; or, if that be not practicable at times, they should divert the current of nervous energy from the organs of the brain which have been unusually exercised, to the consideration of subjects which will employ other organs. This may very well be done by reading a number of short articles on every variety of subject and by various authors, such as we have strung together in the preceding pages. This is very much on the same principle that one set of muscles are rested by the exercise of another set, which allows them to be quiescent.

There are times to all, when the most industrious are utterly indisposed to do a single hand's turn, when the most diligent readers and thinkers lose the power of concentration, and would entirely fail to interest the mind in reading the most exciting history; neither can they go to sleep, which indeed would be the very best thing they could do; and then again, in times of great calamity, or trouble, or despondency, which unfortunately come to all, sooner or later, it will answer an excellent purpose to divert the mind and rest it by reading a variety of short articles, which require no lengthened thought, no special mental effort to take in; even in these cases the reading may sometimes be almost mechanical, yet every now and then a paragraph will be met with which will compel attention more or less; sometimes from its incongruity, its oddity, its fun, its ridiculousness, or its profundity. Some of our weekly exchanges are valuable in this regard, by having half a column or more of miscellanies, brevities, jottings-down, etc.; these afford the means of mental diversion, recreation, and rest, which are of great value in connection with the subject in hand. The *Home Journal* of New-York has a column or two of such reading every week, of great hygienic value. The striking sentences which are met with in reading some new book, and which are industriously penned for the entertainment of its readers, aside from their intrinsic merit, are worth more than money, if used in the ways and at the times referred to in this article.

When a man "don't feel like doing a single thing," he is in danger, because he is very apt, under such circumstances, to dawdle or mope about and do nothing, the very state of mind which the great adversary delights to find, and is sure to take advantage of,

> "For Satan finds some mischief still
> For idle hands to do,"

as the unequaled Isaac Watts has written. Rather than allow perfect idleness under any circumstances read the newspaper with its short and varied articles, even its advertisements, or even an antiquated scrap-book, as a healthful mental diversion, recreation, and rest under the circumstances adverted to.

---

How to make Starch for Shirt-Bosoms. — "Take two ounces of fine gum arabic powder, put it into a pitcher, and pour on a pint or more of boiling water, according to the strength you desire, and then, having covered it, let it stand all night; in the morning pour it carefully from the dregs into a clean bottle, cork it and keep it for use. A table-spoonful of gum-water stirred into a pint of starch made in the usual manner, will give to lawn, either white or printed, a look of newness, when nothing else can restore them after they have been washed."

